# Association Between Volatile Organic Compounds and Circadian Syndrome Among Pre- and Postmenopausal Women

**DOI:** 10.3390/toxics13050328

**Published:** 2025-04-23

**Authors:** Xiaoya Sun, Zhenao Zhang, Jingyi Ren, Huanting Pei, Jie Liu, Bowen Yin, Chongyue Zhang, Rui Wen, Simeng Qiao, Ziyi Wang, Yuxia Ma

**Affiliations:** Hebei Key Laboratory of Environment and Human Health, Department of Nutrition and Food Hygiene, School of Public Health, Hebei Medical University, Shijiazhuang 050017, China

**Keywords:** volatile organic compounds, circadian syndrome, premenopausal, postmenopausal, NHANES

## Abstract

Air pollution is closely associated with the development of multiple metabolic diseases. Circadian syndrome (CircS), as an extended concept of metabolic syndrome (MetS), has been proven to be a better predictor of metabolic diseases than MetS. However, the relationship between volatile organic compounds (VOCs) and CircS in pre- and postmenopausal women remains unclear. This study used data from the National Health and Nutrition Examination Survey (NHANES) 2011–2020, including 520 premenopausal women and 531 postmenopausal women. Generalized linear model (GLM), restricted cubic spline (RCS) model, subgroup analyses, and weighted quantile sum (WQS) model were used to assess the relationship between VOCs and CircS. In addition, sensitivity analyses were performed to evaluate the robustness of the results. Our findings showed that seven VOC metabolites were positively associated with the risk of CircS in postmenopausal women. In premenopausal women, only two VOC metabolites were positively associated with the risk of CircS. The WQS analysis further confirmed that VOC mixtures selected by a least absolute shrinkage and selection operator (LASSO) were significantly associated with an increased risk of CircS in postmenopausal women, with HPMMA identified as the primary contributor to the combined effect. This association was not evident in premenopausal women. Meanwhile, in postmenopausal women, individual urinary VOC metabolites and VOC mixtures were observed to be positively associated with elevated glucose and short sleep. Our results highlighted that VOC exposure was strongly associated with the occurrence of CircS in postmenopausal women. Further research is needed to confirm this conclusion.

## 1. Introduction

Metabolic syndrome (MetS) is a complex disease of metabolic abnormalities, including obesity, hypertension, dyslipidemia, and increased fasting blood glucose. It has emerged as an independent risk factor for cardiovascular diseases (CVDs) and other chronic diseases [1,2]. With the development of social economy and improvements at medical levels, although CVDs have improved in prevention, diagnosis, and treatment, they remain the foremost contributor to mortality and disability worldwide [3]. In recent years, a large amount of evidence has shown that circadian rhythm disruption is closely related to MetS [4,5,6]. Against this background, as an extension of MetS, the concept of circadian syndrome (CircS) has been introduced. CircS adds two additional comorbidities of sleep disorder and depression to MetS, which is a combined concept of metabolic disorder and circadian rhythm disorder. The diagnosis of CircS is based on the presence of at least four conditions, including hypertension, dyslipidemia, increased waist circumference, diabetes, short sleep duration, and depression [7]. Mounting evidence has suggested that CircS is a better predictor of CVDs than MetS [7]. In addition, CircS is also strongly associated with an increased risk of chronic kidney disease, chronic diarrhea, stroke, and many other conditions [8,9,10]. As such, the identification of risk factors for CircS is crucial to prevent a variety of adverse health outcomes.

Menopause is considered to be a critical turning point in the life of a woman’s typical life cycle. The reduction in estrogen levels and the declination in ovarian function for women during their perimenopausal stages lead to an increased risk of developing other diseases, especially endocrine disorders [11,12]. As previously reported in the literature, women exhibit a greater susceptibility to MetS, sleep disorders, and depression compared with men. This susceptibility is markedly exacerbated by the onset of menopause [13,14,15]. Consequently, the identification of risk factors both before and after menopause, alongside the development of efficacious preventive strategies, represents a pivotal research area. And these metabolic disturbances, along with various molecular mechanisms, may also involve an underlying component of circadian rhythm [16]. CircS is a novel concept merging MetS with circadian rhythm disruption, and may be a more sensitive indicator to identify the risk of metabolic diseases before and after menopause.

As urbanization and industrial growth continue to accelerate, air pollution has emerged as a major threat to human health [17]. As an important component of air pollutants, volatile organic compounds (VOCs) exist widely that easily evaporate at normal temperature and pressure. In the general population, air inhalation, food ingestion, and skin contact are the three main ways of exposure to VOCs. Industrial activities, vehicle emissions, and tobacco smoke are major sources of VOCs [18]. Epidemiological and toxicological studies have shown that VOCs can have adverse effects on human health. Studies have shown that exposure to VOCs is strongly associated with altered levels of lung function, cognitive impairment, and an increased risk of various diseases [19,20,21]. Furthermore, an increasing number of research reports claim that environmental pollutants may directly cause circadian rhythm disorders by influencing hormone levels, gene expression, and other pathways [22,23]. However, to the best of our knowledge, evidence regarding the association between VOCs and CircS among pre- and postmenopausal women remains limited. Some cross-sectional studies and animal experiments have investigated the link between VOCs and components of CircS (e.g., obesity, blood pressure, diabetes, depression, and sleep disorders) [24,25,26,27,28]; however, these studies generally lack measurements of key defining factors for CircS and are mostly concentrated in the adult population. Studies on disease risk assessment in menopausal women is still very lacking. In this study, we aimed to investigate the potential association between individual VOCs and mixed VOC exposure and CircS, utilizing data from the National Health and Nutrition Examination Survey (NHANES), with a focus on differences before and after menopause. This will help identify risk factors for metabolic diseases in menopausal women, and provide new insights and epidemiological evidence for their prevention.

## 2. Methods

### 2.1. Study Population

NHANES is a cross-sectional survey conducted by the Centers for Disease Control and Prevention (CDC) that combines interviews with physical examinations to monitor the health and nutritional status of the United States population. The survey was approved by the Review Board of the National Center for Health Statistics Ethics, and all participants provided informed consent. NHANES data information is publicly available (https://wwwn.cdc.gov/nchs/nhanes/Default.aspx, accessed on 21 November 2024). This study used public data from five cycles of NHANES (2011–2012, 2013–2014, 2015–2016, and 2017–2020.03), focusing on women aged 20 and above. Individuals lacking complete data on CircS, urinary VOC metabolites, urinary creatinine, menopausal status, and covariates (i.e., race, education level, poverty income ratio (PIR), marital status, drinker, smoker, and physical activities) were excluded. In addition, given that pregnancy is closely associated with irregular patterns of sleep and various hormonal and metabolic changes, we further excluded pregnant women [29]. The final study included 520 premenopausal women and 531 postmenopausal women (Figure S1).

### 2.2. Menopausal Status

Referring to the previous literature [30], menopausal status was assessed using two inquiries from the NHANES questionnaire as follows: (1) Have you experienced at least one menstrual period in the past 12 months? (2) What is the reason for not having had a menstrual period in the past year? Participants who answered “no” to the first question and “menopause/hysterectomy” to the second question were defined as postmenopausal. And participants who answered “yes” to the first question, or “no” to the first question and “pregnancy”, “breast feeding”, “medical conditions/treatments”, or “other” to the second question, and they were younger than 55 years of age were defined as premenopausal.

### 2.3. Measurement of Urinary VOC Metabolites

Urine samples were professionally collected from participants and then stored at −20 °C until analysis. Urinary VOC metabolites were quantitatively measured by ultra-performance liquid chromatography–electrospray tandem mass spectrometry (UPLC-ESI/MSMS) [31]. Detailed quality control and operational procedures are available on the NHANES website (https://wwwn.cdc.gov/nchs/data/nhanes/public/2015/labmethods/UVOC_UVOCS_I_MET.pdf, accessed on 21 November 2024). The concentrations of VOC metabolites below the limits of detection (LODs) were recorded by LOD/√2. In our study, in order to ensure the validity of the analysis results, we eventually included 15 VOC metabolites with a detection rate of more than 80%: 2-methylhippuric acid (2MHA), 3-methylhippuric acid and 4-methylhippuric acid (3MHA+4MHA), N-acetyl-S-(2-carbamoylethyl)-L-cysteine (AAMA), N-acetyl-S-(N-methylcarbamoyl)-L-cysteine (AMCC), 2-aminothiazoline-4-carboxylic acid (ATCA), N-acetyl-S-(benzyl)-L-cysteine (BMA), N-acetyl-S-(2-carboxyethyl)-L-cysteine (CEMA), N-acetyl-S-(2-cyanoethyl)-L-cysteine (CYMA), N-acetyl-S-(3,4-dihidroxybutyl)-L-cysteine (DHBMA), N-acetyl-S-(2-hydroxypropyl)-L-cysteine (2HPMA), N-acetyl-S- (3-hydroxypropyl)-L-cysteine (3HPMA), N-acetyl-S-(3-hydroxypropyl-1-methyl)-L-cysteine (HPMMA), mandelic acid (MA), N-acetyl-S-(4-hydroxy-2-butenyl)-L-cysteine (MHBMA3), and phenylglyoxylic acid (PGA). Prior to analysis, the effective VOC metabolite concentrations were calibrated by urinary creatinine (Cr) concentrations to avoid inaccuracies in urinary VOC metabolites due to external causes such as metabolic level or water intake, and they were expressed in μg/g Cr [27]. Table S1 shows the parent compounds, abbreviations, LODs, and distributions for each urinary VOC metabolite.

### 2.4. Measurement of Circadian Syndrome

According to previous research [32], CircS diagnoses include components of MetS, short sleep, and depression. MetS was defined according to standards developed by the International Diabetes Federation Task Force [6]. CircS was diagnosed based on the presence of four or more of the following components: elevated waist circumference (≥102 cm in male, ≥88 cm in female), elevated blood pressure (systolic ≥ 130 mmHg or diastolic ≥ 85 mmHg) or use of anti-hypertension medications, elevated fasting glucose (≥100 mg/dL) or taking anti-diabetic pills, elevated triglycerides (≥150 mg/dL) or use of lipid-lowering medications, reduced high-density lipoprotein cholesterol (HDL-C) (<40 mg/dL in male, <50 mg/dL in female) or use of lipid-lowering drugs, short sleep (self-reported < 6 h per day), and depressive symptoms. A person who meets four or more of these seven ingredients was considered CircS [33].

Anthropometric and biochemical measurements were assessed by trained staff. Depressive symptoms were evaluated by the Patient Health Questionnaire (PHQ-9) and were defined as depressive symptoms when participants had a questionnaire score of 10 or greater [34].

### 2.5. Covariates

Based on the current literature [35,36,37], several variables were adjusted as covariates to consider the potential confounders: age, race (Mexican American, other Hispanic, Non-Hispanic White, Non-Hispanic Black, and other), educational level (less than high school, high school, and higher than high school), PIR (<1, 1–3, >3), marital status (married/living with partner, widowed/divorced/separated, never married), drinker, smoker, and physical activity. Drinkers were defined as individuals who consumed a minimum of 12 alcoholic beverages of any kind over the course of a year [38]. Smokers were considered to have smoked more than 100 cigarettes in their life [39]. Physical activity levels were assessed based on the duration of moderate to vigorous physical activity each week and classified as inactivity (<10 min/week) and activity (>10 min/week) [40].

### 2.6. Statistical Analysis

Continuous variables were described by means (standard deviation) or median (interquartile range), while categorical variables were represented by frequency or percentage. For continuous variables with a normal distribution, the independent t-test was used to compare baseline differences between the pre- and postmenopausal groups. For continuous variables with a non-normal distribution, the Mann–Whitney U test was employed. Chi-square tests were utilized for categorical variables. To reduce the effect of extreme values, natural logarithm (ln) transformation of urinary VOC metabolites was performed prior to various analyses. Pearson’s correlation was applied to assess the correlation among multiple VOCs. According to the instructions provided by the NHANES database, we used the subsample weights provided in the VOC samples for weighted analysis. The weights for the new multicycle sample were calculated using the following formulas: (1) Weight = Weight × (2/9.2) for cycles 2011–2012, 2013–2014, and 2015–2016; and (2) Weight = Weight × (3.2/9) for the cycles 2017–2020.

This study used a variety of statistical models to assess the relationships between VOC exposure and CircS before and after menopause. Firstly, we used a weighted multivariable generalized linear model (GLM) to analyze the relationship between individual VOC exposure and CircS. A restricted cubic spline (RCS) regression model was then used to assess the potential nonlinear dose–response associations between VOC metabolites and CircS. Finally, to avoid the collinearity problem, we regressed the model through the least absolute shrinkage and selection operator (LASSO). After screening several key VOCs related to CircS, a weighted quantile sum (WQS) regression model was used to further study the effect of VOC metabolite mixtures on CircS. In the WQS model, we set the ratio of training and verification to 0.4 and 0.6, respectively, and bootstrap to 10,000 times.

In addition, we also performed subgroup analysis and sensitivity analysis to check the stability of the results. Firstly, we assessed whether age, education level, PIR, physical activity, smoker, and drinker had a modifiable effect on the relationship between VOC metabolites and CircS through subgroup analysis. Secondly, the influence of confounding factors was reduced or eliminated by 1:1 propensity score matching (PSM), and the data after PSM were re-analyzed to further verify the accuracy of the results. Finally, we repeated the association analysis after excluding individuals with excessively diluted (Cr < 30 mg/dL) or concentrated (Cr > 300 mg/dL) urine samples.

All analyses were conducted in R (version 4.3.1) and adjusted for all covariates, considering a two-sided *p*-value < 0.05 as significant. The analysis results of WQS model weight plots were generated using the CNSknowall platform (https://cnsknowall.com), a comprehensive web service for data analysis and visualization.

## 3. Results

### 3.1. Baseline Demographic Characteristics

A total of 1051 females were included in this study, with a median age of 50.0 years (quartile 1, quartile 3: 34.0, 62.0 years). Of these participants, 481 (45.8%) were diagnosed with MetS and 356 (33.9%) with CircS. Specifically, of 520 premenopausal women, 141 (27.1%) had MetS and 86 (16.5%) had CircS, while, of 531 postmenopausal women, 340 (64.0%) had been diagnosed with MetS and 270 (50.8%) had been diagnosed with CircS. Table 1 delineates the baseline characteristics of subjects in accordance with menopausal status. Significant differences were observed in all groups except for the group of depression symptoms and short sleep (*p*-value < 0.05). Among these, compared with premenopausal women, postmenopausal women were older and were more likely to be Non-Hispanic White and smoker, had lower educational level and physical activity, were less likely to drink, but had higher PIR and a prevalence of central obesity, elevated glucose, elevated triglycerides, reduced HDL-C, elevated blood pressure, MetS, and CircS. Furthermore, the demographic characteristic information of different NHANES cycles is shown in Table S1.

### 3.2. Distribution and Correlation of Urinary VOC Metabolites

Table S2 displays the distribution of VOC metabolites in urine. Of the 15 VOC metabolites with a detection rate greater than 80%, DHBMA and HPMMA were detected in 100% of participants. Among them, DHBMA exhibited the highest median concentration (299.00 ng/mL), followed by PGA (median concentration: 209.00 ng/mL). The Pearson correlation model was used to represent the correlation between any two urine VOC metabolites. The results are shown in Figure S2, and the Pearson correlation coefficients ranged from 0.05 to 0.90. The VOC metabolites with the highest correlation were MHBMA3 and HPMMA, 2MHA and 3MHA+4MHA, and CYMA and MHBMA3, and the corresponding correlation coefficients were 0.90, 0.88, and 0.85, respectively.

### 3.3. Associations Between Single Urinary VOC Metabolites and CircS Revealed by the Weighted Multiple Linear Regression Model

Table 2 shows the results of weighted GLM between single urinary VOC metabolites and CircS both in pre- and postmenopausal women. After adjusting for all confounders, we found that AAMA, CEMA, CYMA, 3HPMA, HPMMA, MA, and MHBMA3 were significantly positively correlated with an increased risk of CircS in postmenopausal women. Among them, MA had the highest OR (2.000, 95% CI: 1.240 to 3.224), followed by CEMA (OR: 1.736, 95% CI: 1.269 to 2.376) and HPMMA (OR: 1.676, 95% CI: 1.172 to 2.397). However, regarding the association of urinary VOC metabolites with CircS in premenopausal women, only two metabolites were statistically significant: ATCA (OR: 1.658, 95% CI: 1.011 to 2.721) and CEMA (OR: 1.693, 95% CI: 1.144 to 2.504).

### 3.4. Dose–Response Relationship Between Single Urinary VOC Metabolites and CircS Assessed by the RCS

**Figure 1:**
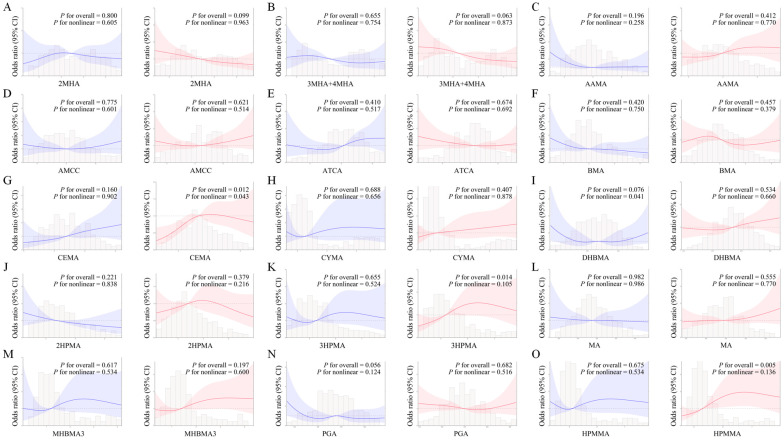
Associations of 15 urinary VOC metabolites, including 2MHA (**A**), 3MHA+4MHA (**B**), AAMA (**C**), AMCC (**D**), ATCA (**E**), BMA (**F**), CEMA (**G**), CYMA (**H**), DHBMA (**I**), 2HPMA (**J**), 3HPMA (**K**), MA (**L**), MHBMA3 (**M**), PGA (**N**), and HPMMA (**O**), and circadian syndrome in a restricted cubic spline model stratified by menopausal status. The multivariate adjusted OR (solid blue line for premenopausal, solid red line for postmenopausal) and 95% CI (shaded area) for associations between urinary VOC metabolites and circadian syndrome. Models were adjusted for age, race, poverty income ratio, smoker, physical activity, education level, drinker, and marriage status. OR—odds ratio; VOCs—volatile organic compounds.

RCS regression was used to show the dose–response relationships between single urinary VOC metabolites and CircS. After adjusting for several potential factors, in postmenopausal women, Figure 1 indicates a nonlinear relationship between CEMA and CircS (*p* for overall < 0.05 and *p* for nonlinear < 0.05). However, in premenopausal women, we only observed a nonlinear relationship between DHBMA and CircS (*p* for overall > 0.05 and *p* for nonlinear < 0.05).

### 3.5. The Key VOC Metabolites Screened by the LASSO Regression Model

**Figure 2:**
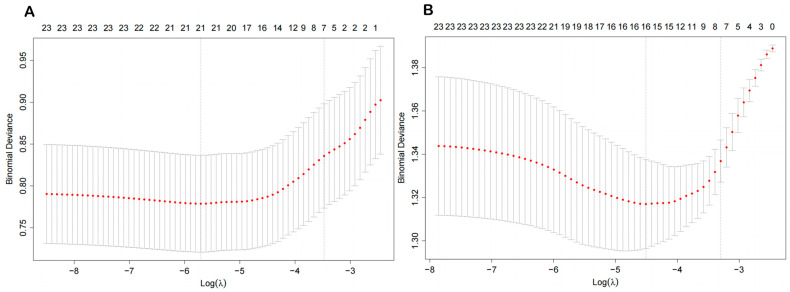
The 10-fold cross-validation curves between log-transformed LASSO penalty parameter (λ) and variables in premenopausal (**A**) and postmenopausal (**B**). The independent variables are ln-transformed 15 VOCs. Covariates include age, race, poverty income ratio, smoker, physical activity, education level, drinker, and marriage status. The left black dotted line is the optimal value of λ at which the minimum binomial deviance is calculated, while the right black dotted line is the value of λ in the simplest model obtained at one standard error of the minimum binomial deviance. LASSO—least absolute shrinkage and selection operator; VOCs—volatile organic compounds.

Pearson correlation analysis revealed a positive correlation between the concentrations of urinary VOC metabolites. To identify VOC metabolites that were more relevant to CircS, we used an adaptive LASSO penalty regression model. Figure 2 shows the correlation between the variation of binomial deviance and the log-transformed penalty parameter (λ) in the LASSO regression model, and the optimal λ values for CircS before and after menopause were 0.00332 (log(λ) = −2.478) and 0.01102 (log(λ) = −1.958), respectively. For CircS in premenopausal women, a total of 14 VOCs were included in the analysis, excluding 3HPMA. For CircS in postmenopausal women, 2MHA, 3MHA+4MHA, ATCA, BMA, CEMA, 2HPMA, MA, HPMMA, and PGA were included. Therefore, we screened 14 and 9 key VOCs associated with CircS in pre- and postmenopausal women, respectively (Figure S3).

### 3.6. Associations of Key Urinary VOC Metabolites with CircS Evaluated by the WQS Model

**Figure 3:**
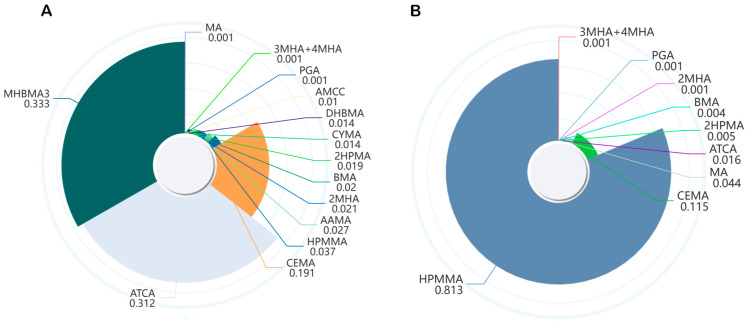
The WQS model weights of screened urinary VOC metabolites on circadian syndrome in premenopausal (**A**) and postmenopausal (**B**) in positive direction. Models were adjusted for age, race, poverty income ratio, smoker, physical activity, education level, drinker, and marriage status. VOC—volatile organic compound; WQS—weighted quantile sum.

By constructing WQS regression models, we evaluated the effects of selected VOC metabolite mixtures on CircS in pre- and postmenopausal women based on the results of LASSO, as shown in Table S3 and Figure 3. When the WQS regression was constrained in the positive direction, the results showed that, after adjusting for all covariates, the WQS index was significantly positively correlated with CircS (OR: 1.103, 95% CI: 1.003 to 1.213) in postmenopausal women, with HPMMA (0.813) being the major contributor to the WQS index, while this relationship was not significant in premenopausal women. When WQS regression was constrained in the negative direction, no significant association between VOC metabolite mixtures and CircS was observed in either premenopausal or postmenopausal women.

### 3.7. Relationship of Urinary VOC Metabolites with CircS Component

**Figure 4:**
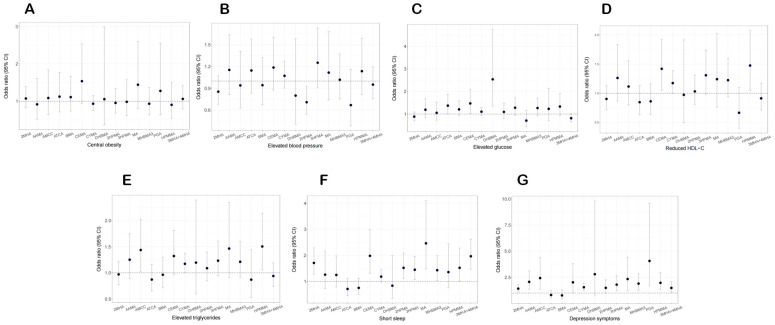
The adjusted odds ratio and its corresponding 95% CI for the seven components of circadian syndrome, including central obesity (**A**), elevated blood pressure (**B**), elevated glucose (**C**), reduced HDL-C (**D**), elevated triglycerides (**E**), short sleep (**F**), and depression symptoms (**G**) with urinary VOC metabolites. Models were adjusted to include age, race, poverty income ratio, smoker, physical activity, education level, drinker, and marriage status. CI—confidence interval; HDL-C—high density lipoprotein cholesterol; OR—odds ratio; VOC—volatile organic compound.

In view of the significant association between individual VOC metabolites, VOC metabolite mixtures, and CircS in postmenopausal women, we further explored the relationship between VOC metabolites and CircS components in postmenopausal women. The relationship between 15 VOC metabolites and CircS components was analyzed by weighted GLM. After adjusting for all confounding factors, we found that HPMMA was positively correlated with reduced HDL-C, elevated triglycerides, short sleep, and depressive symptoms. CEMA was positively associated with elevated glucose, reduced HDL-C, short sleep, and depressive symptoms. Other VOC metabolites also showed positive associations with elevated glucose (DHBMA and ATCA), elevated triglycerides (AMCC and CYMA), short sleep (2MHA, 2HPMA, 3HPMA, MA, MHBMA3, and 3MHA+4MHA), and depressive symptoms (2MHA, AAMA, AMCC, CYMA, 3HPMA, MA, MHBMA3, PGA, and 3MHA+4MHA) (all *p*-value < 0.05). However, no significant positive association was observed between all 15 VOC metabolites and central obesity. In contrast, 2HPMA was significantly inversely associated with elevated blood pressure (Figure 4).

**Figure 5:**
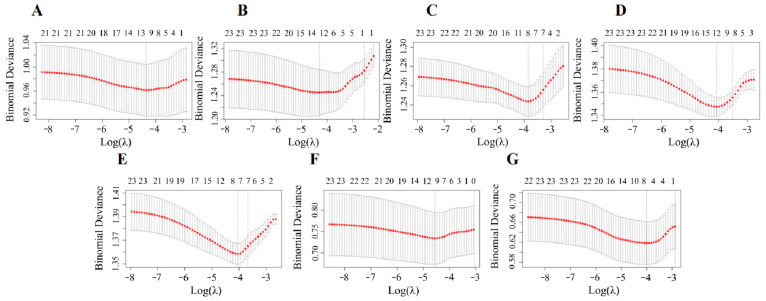
The 10-fold cross-validation curves between log-transformed LASSO penalty parameter (λ) and variables. The independent variables are ln-transformed 15 VOCs. Covariates include age, race, poverty income ratio, smoker, physical activity, education level, drinker, and marriage status. Central obesity (**A**), elevated blood pressure (**B**), elevated glucose (**C**), reduced HDL-C (**D**), elevated triglycerides (**E**), short sleep (**F**), and depression symptoms (**G**) were examined as dependent variables, respectively. The left black dotted line is the optimal value of λ at which the minimum binomial deviance is calculated, while the right black dotted line is the value of λ in the simplest model obtained at one standard error of the minimum binomial deviance. HDL-C—high density lipoprotein cholesterol; LASSO—least absolute shrinkage and selection operator; VOCs—volatile organic compounds.

Considering that highly positive correlations were identified among VOC metabolites, we performed LASSO regression to screen out a set of simple VOC metabolites that were more correlated with CircS components. Figure 5 shows the associations between the variation of binomial deviance and the log-transformed penalty parameter (λ) in LASSO regression; 0.01294 (log(λ) = −1.888), 0.01348 (log(λ) = −1.870), 0.02126 (log(λ) = −1.672), 0.01693 (log(λ) = −1.771), 0.01750 (log(λ) = −1.757), 0.01037 (log(λ) = −1.984), and 0.01828 (log(λ) = −1.738) were selected as the optimal λ values by using central obesity, elevated blood pressure, elevated glucose, reduced HDL-C, elevated triglycerides, short sleep, and depressive symptoms as response variables, respectively. In the best-fit LASSO model, a total of 7, 5, 3, 7, 3, 6, and 5 non-zero coefficient VOC metabolites were more associated with central obesity, elevated blood pressure, elevated glucose, reduced HDL-C, elevated triglycerides, short sleep, and depressive symptoms, respectively (Figure S4).

The correlations between the VOC metabolite mixture index and seven CircS components were analyzed by WQS regression, and the results are shown in Figure S5 and Table S4. The WQS regression results showed that the WQS index was significantly positively correlated with central obesity, elevated glucose, and short sleep, with the OR being 1.158 (95% CI: 1.007 to 1.333), 1.118 (95% CI: 1.011 to 1.237), and 1.310 (95% CI: 1.105 to 1.553), respectively. CEMA (0.448), ATCA (0.592) and 3MHA+4MHA (0.585) had the highest contribution to central obesity, elevated glucose, and short sleep, respectively. However, no significant associations were found between WQS index and elevated blood pressure, reduced HDL-C, elevated triglycerides, and depressive symptoms.

### 3.8. Subgroup Analysis

To further evaluate the consistency of the associations between VOC metabolites and CircS in different subgroups of postmenopausal women, we conducted subgroup analyses and interaction tests stratified by age, drinker, physical activity, smoker, educational level, and PIR. As shown in Tables S5–S10, after adjusting for all confounding factors, the results of different subgroup analyses were consistent with those of the main analysis. And age, drinker, physical activity, smoker, educational level, and PIR did not change the associations between VOC metabolites and CircS.

### 3.9. Sensitivity Analysis

To evaluate the robustness of the association between VOC metabolites and CircS in pre- and postmenopausal women, we first evaluated the association between 15 VOC metabolites and CircS through PSM analysis. Table S11 shows that the results after PSM were consistent with those before matching. Second, the relationship between VOC metabolites and CircS remained strong even after excluding samples from individuals with urine that was either too dilute (Cr < 30 mg/dL) or too concentrated (Cr > 300 mg/dL) (Table S12).

## 4. Discussion

This study used a series of statistical methods to assess the potential association between exposure to VOCs and CircS in pre- and postmenopausal women. For postmenopausal women, results from GLM showed that increased urine levels of AAMA, CEMA, CYMA, 3HPMA, HPMMA, MA, and MHBMA3 were positively associated with the prevalence of CircS. However, only ATCA and CEMA were positively correlated with the occurrence of CircS in premenopausal women. To minimize the potential collinearity of the variables and estimate the combined effects of the VOC mixtures, both the LASSO regression model and the WQS model were utilized. The results showed that mixed VOCs exposure was positively correlated with the risk of CircS in postmenopausal women, with HPMMA accounting for the largest weight, while this relationship was not significant in premenopausal women. This finding implies that postmenopausal women are at a greater risk of developing CircS than premenopausal women, which is consistent with recent research results showing that exposure to air pollution may make postmenopausal women more vulnerable to adverse effects [41].

It is well known that MetS imposes significant socio-economic costs in most countries, and the problem is not well addressed [42]. With the introduction of the CircS concept, it may be more useful to focus on evidence-based prevention of the global burden of noncommunicable diseases (NCDs). There is growing evidence that air pollution disproportionately contributes to the global burden of NCDs [43]. Although the relationship between air pollutants and CircS is still not fully understood, it is worth noting that the association between air pollution and MetS, sleep disorders, and depression has been confirmed by extensive epidemiological evidence [44,45,46]. A study in China showed that sustained exposure to higher concentrations of air pollutants in the Chinese population was linked to a higher prevalence of MetS and its components [44]. An 8-year national cohort study showed that continuous exposure to air pollutants can increase the occurrence of sleep disorders [45]. Meanwhile, the study by Wei F et al. [46] suggested that reducing air pollution was necessary for the disease burden of depression. These research results further support our findings on the health impact of VOCs and highlight the potential risks of air pollution to human health. VOCs, as an important component of air pollutants, are closely related to the occurrence of diseases such as kidney stones, diabetes, CVDs, and depression, which pose serious threats to human health [26,27,47,48]. In addition, menopausal status affecting MetS, sleep duration, and depression have also been reported [12,49,50]. Therefore, a comprehensive assessment of the association between VOCs and CircS among pre- and postmenopausal women has important public health implications. Notably, this is the first study to investigate the associations between single and overall VOC exposure and CircS in a nationally representative sample of pre- and postmenopausal women. Our findings may fill a gap in the understanding of the health hazards of VOC exposure in pre- and postmenopausal women.

Given the novelty of CircS, research on it remains limited. However, some research evidence has suggested an association between VOC exposure and MetS. A study examining the relationship between VOC exposure and MetS and its components found a significant positive association between VOC metabolites and both MetS and its components [51]. Sun J et al. [52] reported that high levels of urinary VOCs in the general adult population were associated with self-reported sleep difficulties among participants. Results from another cross-sectional study suggested that exposure to VOCs may have a negative impact on depression [53]. In this study, given that individuals are often exposed to mixtures of VOCs in their daily lives, focusing on only one VOC may lead to potential bias [54]. Therefore, in addition to examining the effects of individual VOCs on CircS, we also used WQS regression to study the combined effects of mixed VOCs, aiming to more accurately portray the complexity of multiple exposures in real-world scenarios. The results showed that both individual VOC and mixed VOC exposure had a greater effect on CircS in postmenopausal women. This may be attributed to the decreased level of estrogen in postmenopausal women, which significantly reduces the protective effect of women, thus making postmenopausal women more prone to dyslipidemia and weight gain than premenopausal women [55]. Therefore, exposure to VOCs in postmenopausal women deserves our special attention.

Furthermore, identifying critical toxic VOCs has significant implications for health promotion. Notably, for postmenopausal women, the crotonaldehyde metabolite HPMMA, the acrolein metabolite CEMA, the cyanide metabolite ATCA, and the xylene metabolite 3MHA+4MHA were the major contributors to CircS, central obesity, elevated glucose, and short sleep, respectively. Crotonaldehyde, as an unsaturated aldehyde, is significantly related to systolic blood pressure [25]. Acrolein increases oxidative stress (OS) and apoptosis, alters pro-inflammatory mediators, and alters cell signaling, thereby causing or inducing diseases in various organs [56]. Cyanide mainly interferes with cellular respiratory processes, causing many diseases and even death [57]. Exposure to xylene, a cyclic hydrocarbon, was strongly linked to an increased risk of MetS [58]. However, exposure to crotonaldehyde, acrolein, cyanide, and xylene has not previously been reported to be associated with CircS and its components. Therefore, these adverse effects on postmenopausal women are of concern.

Despite the above findings, the underlying mechanisms are still not fully understood. We can consider several possible factors contributing to these associations. VOCs, as key components of lipid peroxidation and insulin resistance, can induce inflammation and OS [59,60,61,62]. Elevated insulin resistance and lipid peroxidation are associated with obesity, diabetes, hyperlipidemia, sleep deprivation, and depression [63,64,65,66,67]. In addition, insulin resistance and lipid metabolism disorders are a serious complication in menopausal women [68,69]. Therefore, the above factors may collectively promote the occurrence of CircS in postmenopausal women. However, while these may explain the potential association between VOCs and CircS, few experiments have directly investigated whether VOCs cause CircS in postmenopausal women through these mechanisms. Therefore, further experimental studies are needed to fully elucidate the underlying mechanism.

Our research has several advantages. Firstly, this is the first study in the United States to investigate the association between VOCs and CircS in pre- and postmenopausal women. Furthermore, the NHANES data we used were collected with rigorous collection procedures. Second, we conducted a comprehensive assessment of the associations between single VOC metabolite and mixed VOC exposure with CircS by using multiple statistical methods, which improved the stability of our findings. At the same time, we adjusted for a large number of potential confounding factors, including demographic and lifestyle factors. Finally, subgroup analysis and sensitivity analysis were also conducted to better use the data to reveal the underlying truth. However, there are limitations to this study that need to be discussed. First, due to the fact that many covariates are only available at baseline, we cannot capture confounders’ changes over time. Second, urinary VOCs are measured based on a single point in time, which makes it challenging to assess long-term VOC exposure levels. Thirdly, self-reported menopausal status may influence the classification of participants. Due to the limitations of NHANES data, future studies should further refine the assessment methods for menopausal status. Moreover, considering the influence of factors such as radiotherapy on hormone levels, these factors should be taken into account in future research. In addition, the results of this study may be representative only of United States residents, and further research should be conducted to verify the generality of the results to other populations. Finally, this was a cross-sectional study that cannot provide causal relationships. The adverse effects of VOCs on CircS still need to be further validated in cell, animal, and randomized controlled studies.

## 5. Conclusions

In conclusion, our research indicated that exposure to certain single VOC metabolites and VOC metabolites mixture were positively associated with CircS in postmenopausal women. This association was not evident in premenopausal women. Notably, HPMMA, a metabolite of crotonaldehyde, was identified as a major contributor to CircS. In addition, in postmenopausal, certain individual VOC metabolites and VOC metabolite mixtures had positive correlation with elevated glucose and short sleep, with ATCA and 3MHA+4MHA being identified as the most significant chemical for elevated glucose and short sleep, respectively. The results of our study offer novel insights into assessing exposure to VOCs associated with CircS, with the aim of providing public awareness. Further basic experiments or randomized controlled trials are needed to verify our results.

## Figures and Tables

**Table 1:** Study population characteristics stratified by menopausal status, NHANES 2011–2020 (n = 1051).

Variable	Overall (n = 1051)	Premenopausal (n = 520)	Postmenopausal (n = 531)	*p*-Value
Age (years), median (Q1, Q3)	50.0 (34.0, 62.0)	34.5 (27.0, 43.8)	62.0 (55.0, 70.0)	<0.001
Race, n (%)				0.008
Mexican American	119 (11.3)	61 (11.7)	58 (10.9)	
Other Hispanic	114 (10.8)	56 (10.8)	58 (10.9)	
Non-Hispanic White	406 (38.6)	178 (34.2)	228 (42.9)	
Non-Hispanic Black	253 (24.1)	128 (24.6)	125 (23.5)	
Other	159 (15.1)	97 (18.7)	62 (11.7)	
Educational level, n (%)				<0.001
Below high school	183 (17.4)	75 (14.4)	108 (20.3)	
High school	226 (21.5)	96 (18.5)	130 (24.5)	
Above high school	642 (61.1)	349 (67.1)	293 (55.2)	
Marital status, n (%)				0.006
Married/living with partner	560 (53.3)	300 (57.7)	260 (49.0)	
Widowed/divorced/separated	317 (30.2)	134 (25.8)	183 (34.5)	
Never married	174 (16.6)	86 (16.5)	88 (16.6)	
Physical activity, n (%)				<0.001
Active	772 (73.5)	411 (79.0)	361 (68.0)	
Inactive	279 (26.5)	109 (21.0)	170 (32.0)	
Drinker, n (%)				<0.001
Yes	598 (56.9)	338 (65.0)	260 (43.5)	
No	453 (43.1)	182 (35.0)	271 (51.0)	
Smoker, n (%)				0.002
Yes	377 (35.9)	163 (31.3)	214 (40.3)	
No	674 (64.1)	357 (68.7)	317 (59.7)	
Poverty income ratio, n (%)				0.002
<1.0	243 (23.1)	144 (27.7)	99 (18.6)	
1.0–3.0	424 (40.3)	197 (37.9)	227 (42.7)	
>3.0	384 (36.5)	179 (34.4)	205 (38.6)	
Central obesity, n (%)				<0.001
Yes	757 (72.0)	327 (62.9)	430 (81.0)	
No	294 (28.0)	193 (37.1)	101 (19.0)	
Elevated glucose, n (%)				<0.001
Yes	527 (50.1)	175 (33.7)	352 (66.3)	
No	524 (49.9)	345 (66.3)	179 (33.7)	
Elevated triglycerides, n (%)				<0.001
Yes	362 (34.4)	82 (15.8)	280 (52.7)	
No	689 (65.6)	438 (84.2)	251 (47.3)	
Reduced HDL-C, n (%)				<0.001
Yes	487 (46.3)	185 (35.6)	302 (56.9)	
No	564 (53.7)	335 (64.4)	229 (43.1)	
Elevated blood pressure, n (%)				<0.001
Yes	446 (42.4)	106 (20.4)	340 (64.0)	
No	605 (57.6)	414 (79.6)	191 (36.0)	
Depression symptoms, n (%)				0.750
Yes	108 (10.3)	55 (10.6)	53 (10.0)	
No	943 (89.7)	465 (89.4)	478 (90.0)	
Short sleep, n (%)				0.753
Yes	134 (12.7)	68 (13.1)	66 (12.4)	
No	917 (87.3)	452 (86.9)	465 (87.6)	
Metabolic syndrome, n (%)				<0.001
Yes	481 (45.8)	141 (27.1)	340 (64.0)	
No	570 (54.2)	379 (72.9)	191 (36.0)	
Circadian syndrome, n (%)				<0.001
Yes	356 (33.9)	86 (16.5)	270 (50.8)	
No	695 (66.1)	434 (83.5)	261 (49.2)	

HDL-C—high density lipoprotein cholesterol.

**Table 2:** Associations between single urinary VOC metabolites and circadian syndrome stratified by menopausal status.

VOCs	Premenopausal		Postmenopausal	
	OR (95% CI)	*p*-Value	OR (95% CI)	*p*-Value
2MHA	1.117 (0.882, 1.416)	0.351	0.877 (0.688, 1.119)	0.284
3MHA+4MHA	0.964 (0.749, 1.242)	0.774	0.929 (0.708, 1.220)	0.589
AAMA	0.952 (0.540, 1.677)	0.861	1.436 (1.007, 2.050)	0.046
AMCC	1.172 (0.731, 1.880)	0.502	1.308 (0.913, 1.876)	0.140
ATCA	1.658 (1.011, 2.721)	0.045	0.924 (0.681, 1.253)	0.603
BMA	0.820 (0.561, 1.199)	0.299	0.939 (0.710, 1.242)	0.654
CEMA	1.693 (1.144, 2.504)	0.009	1.736 (1.269, 2.376)	<0.001
CYMA	1.153 (0.951, 1.398)	0.143	1.256 (1.048, 1.505)	0.015
DHBMA	0.825 (0.297, 2.291)	0.707	1.358 (0.646, 2.856)	0.412
2HPMA	0.818 (0.520, 1.288)	0.379	1.053 (0.819, 1.353)	0.682
3HPMA	1.177 (0.779, 1.776)	0.431	1.528 (1.141, 2.047)	0.005
HPMMA	1.245 (0.811, 1.912)	0.309	1.676 (1.172, 2.397)	0.006
MA	1.136 (0.716, 1.801)	0.582	2.000 (1.240, 3.224)	0.005
MHBMA3	1.118 (0.788, 1.586)	0.523	1.350 (1.004, 1.814)	0.047
PGA	0.603 (0.322, 1.130)	0.112	0.885 (0.512, 1.528)	0.654

Models were adjusted for age, race, poverty income ratio, smoker, physical activity, education level, drinker, and marriage status. CI—confidence interval; OR—odds ratio; VOCs—volatile organic compounds.

## Data Availability

Data will be made available on request.

## Supplementary Materials

The following supporting information can be downloaded at: https://www.mdpi.com/article/10.3390/toxics13050328/s1.
