# Supplementary material for: Association Between Volatile Organic Compounds and Circadian Syndrome Among Pre- and Postmenopausal Women
Source: Toxics. 2025 Apr 23;13(5):328. doi: 10.3390/toxics13050328 (PMC12115961; doi:10.3390/toxics13050328)
Supplement: Supplementary file 1 [file toxics-13-00328-s001.zip › toxics-3577158-supplementary.pdf]

Association between volatile organic compounds and circadian syndrome among pre- and postmenopausal women

Xiaoya Sun[a, 1], Zhenao Zhang[a, 1], Jingyi Ren[a], Huanting Pei[a], Jie Liu[a], Bowen Yin[a], Chongyue Zhang[a], Rui Wen[a], Simeng Qiao[a], Ziyi Wang[a], Yuxia Ma[a, *]

[a]Department of Nutrition and Food Hygiene, School of Public Health, Hebei Medical University, Hebei Key Laboratory of Environment and Human Health, Shijiazhuang, 050017, China

[1]Co-first authors

[*]Corresponding authors:

Yuxia Ma, PHD. Department of Nutrition and Food Hygiene, School of Public Health, Hebei Medical University, Hebei Key Laboratory of Environment and Human Health, Shijiazhuang, 050017, China. E-mail: mayuxia@hebmu.edu.cn.

This file includes:

Table S1. Study population characteristics stratified by NHANES cycle (n=1051).

Table S2. The concentrations of urinary VOC metabolites (ng/mL).

Table S3. Associations of WQS regression index with circadian syndrome stratified by menopausal status.

Table S4. Associations of WQS regression index with seven components of circadian syndrome in postmenopausal women.

Table S5. Subgroup analysis of the associations between urinary VOC metabolites and circadian syndrome stratified by age in postmenopausal women.

Table S6. Subgroup analysis of the association between urinary VOC metabolites and circadian syndrome stratified by drinker in postmenopausal women.

Table S7. Subgroup analysis of the association between urinary VOC metabolites and circadian syndrome stratified by PA in postmenopausal women.

Table S8. Subgroup analysis of the association between urinary VOC metabolites and circadian syndrome stratified by smoker in postmenopausal women.

Table S9. Subgroup analysis of the association between urinary VOC metabolites and circadian syndrome stratified by education level in postmenopausal women.

Table S10. Subgroup analysis of the association between urinary VOC metabolites and circadian syndrome stratified by poverty income ratio in postmenopausal women.

Table S11. Sensitivity analysis of the relationship between urinary VOC metabolites and circadian syndrome by using PSM.

Table S12. Sensitivity analysis of the relationship between urinary VOCs and circadian syndrome after excluding individuals with urinary creatinine >300 mg/L or urinary creatinine <30 mg/L.

Fig. S1. Flowchart of participants included in this study.

Fig. S2. Pearson correlations among the urinary VOC metabolites.

Fig. S3. Shrinkage coefficient curves between the log-transformed LASSO penalty parameter (λ) and the variates in premenopausal (A) and postmenopausal (B).

Fig. S4. Shrinkage coefficient curves between the log-transformed LASSO penalty parameter (λ) and the variates.

Fig. S5. The WQS model weights of screened urinary VOC metabolites on Central obesity (A), Elevated blood pressure (B), Elevated glucose (C), Reduced HDL-C (D), Elevated triglycerides (E), Short sleep(F), Depression symptoms (G).

Table S1. Study population characteristics stratified by NHANES cycle (n=1051).

Variable	2011-2012 (n =248)	2013-2014 (n =194)	2015-2016 (n=197)	2017-2020 (n = 412)	*P*-value
Age (years), median (Q1, Q3)	50.0 (33.0, 64.0)	50.0 (34.0, 62.0)	50.0 (35.0, 64.0)	49.0 (36.0, 60.0)	0.952
Race, n (%)					< 0.001
Mexican American	18 (7.3)	20 (10.3)	29 (14.7)	52 (12.6)	
Other Hispanic	28 (11.3)	22 (11.3)	32 (16.2)	32 (7.8)	
Non-Hispanic White	103 (41.5)	92 (47.4)	71 (36.0)	140 (34.0)	
Non-Hispanic Black	65 (26.2)	44 (22.7)	34 (17.3)	110 (26.7)	
Other	34 (13.7)	16 (8.2)	31 (15.7)	78 (18.9)	
Educational level, n (%)					0.072

Below high school	48 (19.4)	30 (15.5)	43 (21.8)	62 (15.0)	
High school	45 (18.1)	35 (18.0)	41 (20.8)	105 (25.5)	
Above high school	155 (62.5)	129 (66.5)	113 (57.4)	245 (59.5)	
Marital status, n (%)					0.627
Married/living with partner	124 (50.0)	96 (49.5)	107 (54.3)	233 (56.6)	
Widowed/divorced/separated	82 (33.1)	62 (32.0)	59 (29.9)	114 (27.7)	
Never married	42 (16.9)	36 (18.6)	31 (15.7)	65 (15.8)	
Physical activity, n (%)					0.468
Activity	189 (76.2)	142 (73.2)	137 (69.5)	304 (73.8)	
Inactive	59 (23.8)	52 (26.8)	60 (30.5)	108 (26.2)	

Drinker, n (%)					< 0.001
Yes	165 (66.5)	121 (62.4)	122 (61.9)	190 (46.1)	
No	83 (33.5)	73 (37.6)	75 (38.1)	222 (53.9)	
Smoker, n (%)					0.916
Yes	90 (36.3)	67 (34.5)	68 (34.5)	152 (36.9)	
No	158 (63.7)	127 (65.5)	129 (65.5)	260 (63.1)	
Poverty income ratio, n (%)					0.539
< 1.0	66 (26.6)	47 (24.2)	40 (20.3)	90 (21.8)	
1.0-3.0	92 (37.1)	81 (41.8)	88 (44.7)	163 (39.6)	
> 3.0	90 (36.3)	66 (34.0)	69 (35.0)	159 (38.6)	

NHANES, National Health and Nutrition Examination Survey.

Table S2. The concentrations of urinary VOC metabolites (ng/mL).

VOC metabolites	Abbreviation	Parent Compound	LOD (ng/mL) [a]	Detection Rate (%)	Concentration [b]	Concentration [c]
2-methylhippuric acid	2MHA	Xylene	5.000	88.5%	27.90 (10.75, 73.20)	63.56 (103.59)
3- methylhippuric acid & 4- methylhippuric acid	3MHA+4MHA	Xylene	8.000	99.6%	190.00 (72.40, 562.00)	444.03 (726.88)
N-acetyl-S-(2-carbamoylethyl)-L-cysteine	AAMA	Acrylamide	2.200	99.9%	48.00 (24.25, 99.90)	87.34 (137.98)
N-acetyl-S-(N-methylcarbamoyl)-L-cysteine	AMCC	N, N-Dimethylformamide	6.260	99.9%	153.00 (74.40, 313.50)	301.02 (1457.79)
2-aminothiazoline-4-carboxylic acid	ATCA	Cyanide	15.000	96.4%	147.00 (74.05, 266.00)	208.26 (217.31)
N-acetyl-S-(benzyl)-L-cysteine	BMA	Toluene	0.500	99.8%	6.73 (3.50, 13.95)	15.47 (42.87)
N-acetyl-S-(2-carboxyethyl)-L-cysteine	CEMA	Acrolein	6.960	99.0%	83.90 (45.90, 169.00)	137.11 (162.70)
N-acetyl-S-(2-cyanoethyl)-L-cysteine	CYMA	Acrylonitrile	0.500	83.4%	1.47 (0.73, 5.90)	39.76 (119.78)
N-acetyl-S-(3, 4-dihidroxybutyl)-L-cysteine	DHBMA	1, 3-Butadiene	5.250	100.0%	299.00 (168.50, 462.00)	349.46 (240.35)
N-acetyl-S-(2-hydroxypropyl)-L-cysteine	2HPMA	Propylene oxide	0.791	95.3%	28.60 (14.90, 55.75)	62.16 (161.01)
N-acetyl-S- (3-hydroxypropyl)-L-cysteine	3HPMA	Acrolein	13.000	99.9%	172.00 (93.95, 371.00)	402.58 (828.20)
N-acetyl-S-(3-hydroxypropyl-1-methyl)-L-cysteine	HPMMA	Crotonaldehyde	1.700	100.0%	180.00 (104.50, 323.00)	402.26 (776.86)
Mandelic acid	MA	Styrene	12.000	99.0%	122.00 (71.45, 213.50)	169.84 (164.88)
N-acetyl-S-(4-hydroxy-2-butenyl)-L-cysteine	MHBMA3	1, 3-Butadiene	0.600	96.3%	3.64 (1.97, 7.25)	10.47 (23.18)
Phenylglyoxylic acid	PGA	Ethylbenzene, styrene	12.000	99.9%	209.00 (124.00, 333.00)	264.75 (219.90)

[a] LOD: Limit of Detection.

[b] Median (quartile 1, quartile 3).

[c] Mean (standard deviation).

Table S3. Associations of WQS regression index with circadian syndrome stratified by menopausal status [a].

**WQS index**		**OR**	**95% CI**	***P*-value**
Premenopausal	Positive	1.140	(0.951, 1.366)	0.157
	Negative	0.961	(0.799, 1.156)	0.671
Postmenopausal	Positive	1.103	(1.003, 1.213)	**0.043**
	Negative	0.904	(0.803, 1.018)	0.097

[a] Models were adjusted for age, race, poverty income ratio, smoker, physical activity, education level, drinker and marriage status. CI, confidence interval; OR, odds ratio; WQS, weighted quantile sum.

Table S4. Associations of WQS regression index with seven components of circadian syndrome in postmenopausal women [a].

WQS index	OR	95% CI	*P*-value
Central obesity	1.158	(1.007, 1.333)	**0.039**
Elevated blood pressure	1.032	(0.920, 1.157)	0.595
Elevated glucose	1.118	(1.011, 1.237)	**0.030**
Reduced HDL-C	1.085	(0.973, 1.211)	0.144
Elevated triglycerides	1.057	(0.964, 1.159)	0.239
Short sleep	1.310	(1.105, 1.553)	**0.002**
Depression symptoms	1.094	(0.881, 1.359)	0.418

[a] Models were adjusted for age, race, poverty income ratio, smoker, physical activity, education level, drinker and marriage status. CI, confidence interval; OR, odds ratio; WQS, weighted quantile sum.

Table S5. Subgroup analysis of the associations between urinary VOC metabolites and circadian syndrome stratified by age in postmenopausal women [a].

VOCs	Age <60		Age ≥60		*P* for interaction
	OR (95% CI)	*P*-value	OR (95% CI)	*P*-value	
2MHA	0.993 (0.708, 1.394)	0.967	0.770 (0.588, 1.009)	0.058	0.233
3MHA+4MHA	1.257 (0.892, 1.773)	0.182	0.776 (0.572, 1.051)	0.098	0.046
AAMA	1.695 (0.894, 3.213)	0.102	1.208 (0.764, 1.908)	0.409	0.524
AMCC	1.324 (0.756, 2.319)	0.311	1.264 (0.853, 1.873)	0.235	0.617
ATCA	0.711 (0.450, 1.125)	0.139	1.079 (0.720, 1.618)	0.706	0.212
BMA	1.087 (0.653, 1.811)	0.739	0.831 (0.563, 1.228)	0.343	0.516
CEMA	1.762 (1.017, 3.053)	0.044	1.825 (1.226, 2.718)	0.004	0.917
CYMA	1.454 (1.124, 1.881)	0.006	1.121 (0.899, 1.396)	0.301	0.174
DHBMA	1.311 (0.546, 3.151)	0.530	1.225 (0.457, 3.286)	0.679	0.893
2HPMA	0.947 (0.626, 1.434)	0.790	1.104 (0.806, 1.513)	0.527	0.610
3HPMA	1.354 (0.873, 2.102)	0.167	1.683 (1.159, 2.446)	0.008	0.575
HPMMA	1.637 (1.025, 2.617)	0.040	1.985 (1.197, 3.293)	0.009	0.511
MA	1.564 (0.695, 3.517)	0.267	2.063 (1.204, 3.537)	0.010	0.488
MHBMA3	1.385 (0.919, 2.089)	0.115	1.310 (0.930, 1.846)	0.119	0.702
PGA	1.100 (0.482, 2.509)	0.814	0.689 (0.348, 1.364)	0.276	0.279

[a] Models were adjusted for race, poverty income ratio, smoker, physical activity, education level, drinker and marriage status. CI, confidence interval; OR, odds ratio; VOCs, volatile organic compounds.

Table S6. Subgroup analysis of the association between urinary VOC metabolites and circadian syndrome stratified by drinker in postmenopausal women [a].

VOCs	Drinker		Non-drinker		*P* for interaction
	OR (95% CI)	*P*-value	OR (95% CI)	*P*-value	
2MHA	0.806 (0.582, 1.118)	0.189	1.072 (0.729, 1.575)	0.717	0.271
3MHA+4MHA	0.864 (0.596, 1.252)	0.427	1.057 (0.737, 1.515)	0.755	0.310
AAMA	1.418 (0.868, 2.315)	0.157	1.718 (0.914, 3.229)	0.090	0.609
AMCC	1.350 (0.799, 2.282)	0.253	1.281 (0.747, 2.196)	0.357	0.694
ATCA	0.708 (0.441, 1.136)	0.147	1.359 (0.856, 2.158)	0.186	0.091
BMA	0.848 (0.567, 1.267)	0.409	1.262 (0.865, 1.842)	0.219	0.178
CEMA	1.626 (1.092, 2.420)	0.018	2.185 (1.126, 4.240)	0.022	0.520
CYMA	1.199 (0.950, 1.514)	0.122	1.491 (1.119, 1.986)	0.008	0.415
DHBMA	1.377 (0.512, 3.703)	0.514	1.257 (0.465, 3.398)	0.642	0.815
2HPMA	1.072 (0.762, 1.508)	0.681	0.969 (0.663, 1.418)	0.868	0.783
3HPMA	1.543 (1.029, 2.316)	0.037	1.891 (1.114, 3.212)	0.030	0.662
HPMMA	1.663 (1.018, 2.717)	0.043	1.754 (1.062, 2.897)	0.020	0.523
MA	2.380 (1.239, 4.572)	0.011	1.755 (0.861, 3.580)	0.117	0.749
MHBMA3	1.247 (0.829, 1.878)	0.279	1.664 (1.095, 2.529)	0.019	0.395
PGA	0.681 (0.313, 1.478)	0.320	1.265 (0.600, 2.667)	0.524	0.227

[a] Models were adjusted for age, race, poverty income ratio, smoker, physical activity, education level and marriage status. CI, confidence interval; OR, odds ratio; VOCs, volatile organic compounds.

Table S7. Subgroup analysis of the association between urinary VOC metabolites and circadian syndrome stratified by PA in postmenopausal women [a].

VOCs	Activity		Inactive		*P* for interaction
	OR (95% CI)	*P*-value	OR (95% CI)	*P*-value	
2MHA	0.972 (0.739, 1.279)	0.835	0.740 (0.468, 1.169)	0.183	0.331
3MHA+4MHA	1.013 (0.747, 1.375)	0.930	0.851 (0.524, 1.382)	0.491	0.546
AAMA	1.516 (0.941, 2.444)	0.085	1.437 (0.822, 2.514)	0.189	0.545
AMCC	1.280 (0.795, 2.061)	0.301	1.396 (0.728, 2.677)	0.295	0.335
ATCA	1.025 (0.706, 1.488)	0.893	0.534 (0.295, 0.968)	0.040	0.087
BMA	0.843 (0.581, 1.224)	0.359	1.236 (0.774, 1.973)	0.353	0.367
CEMA	1.792 (1.176, 2.732)	0.008	1.801 (0.847, 3.828)	0.118	0.726
CYMA	1.331 (1.060, 1.672)	0.015	1.112 (0.811, 1.524)	0.488	0.873
DHBMA	1.245 (0.545, 2.844)	0.594	1.852 (0.408, 8.417)	0.402	0.425
2HPMA	1.055 (0.775, 1.436)	0.727	1.052 (0.625, 1.772)	0.840	0.653
3HPMA	1.948 (1.299, 2.923)	0.002	0.890 (0.503, 1.574)	0.671	0.108
HPMMA	2.048 (1.269, 3.304)	0.004	1.115 (0.611, 2.038)	0.707	0.487
MA	2.133 (1.208, 3.767)	0.011	1.959 (0.670, 5.733)	0.204	0.699
MHBMA3	1.529 (1.037, 2.254)	0.033	1.074 (0.688, 1.675)	0.739	0.780
PGA	0.861 (0.426, 1.739)	0.669	0.971 (0.372, 2.535)	0.950	0.608

[a] Models were adjusted for age, race, poverty income ratio, smoker, education level, drinker and marriage status. CI, confidence interval; OR, odds ratio; PA, physical activity; VOCs, volatile organic compounds.

Table S8. Subgroup analysis of the association between urinary VOC metabolites and circadian syndrome stratified by smoker in postmenopausal women [a].

VOCs	Smoker		Non-smoker		*P* for interaction
	OR (95% CI)	*P*-value	OR (95% CI)	*P*-value	
2MHA	0.755 (0.570, 1.002)	0.052	0.689 (0.499, 0.952)	0.025	0.195
3MHA+4MHA	0.747 (0.540, 1.032)	0.076	0.756 (0.521, 1.097)	0.136	0.434
AAMA	1.241 (0.778, 1.981)	0.355	1.529 (0.843, 2.771)	0.156	0.717
AMCC	1.230 (0.852, 1.775)	0.261	1.386 (0.839, 2.289)	0.195	0.839
ATCA	1.098 (0.733, 1.646)	0.641	1.186 (0.792, 1.774)	0.396	0.097
BMA	0.832 (0.563, 1.228)	0.344	1.261 (0.866, 1.835)	0.219	0.038
CEMA	1.761 (1.228, 2.524)	0.003	1.875 (1.148, 3.063)	0.014	0.448
CYMA	1.120 (0.914, 1.372)	0.266	1.195 (0.722, 1.979)	0.477	0.850
DHBMA	1.231 (0.469, 3.231)	0.665	1.200 (0.404, 3.564)	0.735	0.985
2HPMA	1.123 (0.810, 1.556)	0.477	0.832 (0.570, 1.215)	0.331	0.069
3HPMA	1.694 (1.221, 2.350)	0.002	1.614 (0.809, 3.218)	0.168	0.965
HPMMA	1.919 (1.216, 3.030)	0.006	1.559 (0.563, 4.319)	0.382	0.992
MA	2.181 (1.319, 3.607)	0.003	3.400 (1.670, 6.921)	0.001	0.102
MHBMA3	1.296 (0.938, 1.790)	0.112	1.341 (0.605, 2.972)	0.458	0.944
PGA	0.693 (0.361,1.330)	0.262	0.824 (0.376,1.807)	0.620	0.849

[a] Models were adjusted for age, race, poverty income ratio, physical activity, education level, drinker and marriage status. CI, confidence interval; OR, odds ratio; VOCs, volatile organic compounds.

Table S9. Subgroup analysis of the association between urinary VOC metabolites and circadian syndrome stratified by educational level in postmenopausal women [a].

VOCs	Below high school		High school		Above high school		*P* for interaction
	OR (95% CI)	*P*-value	OR (95% CI)	*P*-value	OR (95% CI)	*P*-value	
2MHA	1.261 (0.698, 2.276)	0.392	1.122 (0.636, 1.978)	0.669	0.739 (0.539, 1.013)	0.060	0.195
3MHA+4MHA	1.053 (0.511, 2.167)	0.873	1.017 (0.613, 1.689)	0.942	0.879 (0.615, 1.256)	0.468	0.434
AAMA	1.450 (0.781, 2.691)	0.203	1.827 (0.705, 4.734)	0.194	1.412 (0.876, 2.276)	0.151	0.717
AMCC	1.540 (0.531, 4.469)	0.377	1.727 (0.676, 4.414)	0.230	1.258 (0.801, 1.977)	0.311	0.839
ATCA	0.867 (0.381, 1.973)	0.700	1.333 (0.757, 2.348)	0.292	0.876 (0.576, 1.332)	0.527	0.097
BMA	0.790 (0.419, 1.488)	0.415	1.382 (0.496, 3.850)	0.507	0.934 (0.619, 1.409)	0.739	0.038
CEMA	2.362 (1.001, 5.575)	0.050	2.030 (0.889, 4.638)	0.087	1.762 (1.135, 2.736)	0.013	0.448
CYMA	0.995 (0.728, 1.362)	0.974	1.387 (0.926, 2.077)	0.104	1.264 (0.997, 1.602)	0.053	0.850
DHBMA	1.418 (0.197, 10.197)	0.694	2.123 (0.501, 8.996)	0.280	1.310 (0.542, 3.168)	0.540	0.985
2HPMA	1.046 (0.304, 3.600)	0.935	1.035 (0.520, 2.062)	0.915	1.091 (0.765, 1.556)	0.624	0.069

3HPMA	1.804 (0.791, 4.114)	0.138	1.351 (0.654, 2.791)	0.386	1.700 (1.156, 2.500)	0.008	0.965
HPMMA	2.095 (0.851, 5.162)	0.095	1.644 (0.782, 3.453)	0.172	1.662 (1.047, 2.639)	0.032	0.992
MA	1.783 (0.496, 6.419)	0.328	1.609 (0.517, 5.003)	0.382	2.574 (1.429, 4.637)	0.002	0.102
MHBMA3	1.508 (0.769, 2.957)	0.197	1.540 (0.815, 2.909)	0.166	1.305 (0.866, 1.968)	0.197	0.944
PGA	0.750 (0.239, 2.350)	0.577	0.746 (0.263, 2.117)	0.554	1.014 (0.489, 2.105)	0.969	0.849

[a] Models were adjusted for age, race, poverty income ratio, smoker, physical activity, drinker and marriage status. CI, confidence interval; OR, odds ratio; VOCs, volatile organic compounds.

Table S10. Subgroup analysis of the association between urinary VOC metabolites and circadian syndrome stratified by poverty income ratio in postmenopausal women [a].

VOCs	Poverty income ratio < 1.0		Poverty income ratio 1.0-3.0		Poverty income ratio > 3.0		*P* for interaction
	OR (95% CI)	*P*-value	OR (95% CI)	*P*-value	OR (95% CI)	*P*-value	
2MHA	1.696 (0.943, 3.053)	0.072	0.969 (0.679, 1.383)	0.859	0.729 (0.504, 1.056)	0.091	0.037
3MHA+4MHA	1.443 (0.790, 2.635)	0.202	0.906 (0.656, 1.250)	0.534	0.897 (0.608, 1.323)	0.568	0.295
AAMA	1.997 (0.970, 4.112)	0.058	1.724 (0.921, 3.229)	0.086	1.311 (0.756, 2.273)	0.319	0.726
AMCC	2.738 (1.101, 6.811)	0.034	1.225 (0.702, 2.139)	0.463	1.319 (0.721, 2.414)	0.353	0.432
ATCA	1.060 (0.585, 1.922)	0.828	0.891 (0.582, 1.364)	0.585	0.962 (0.561, 1.650)	0.885	0.688
BMA	1.194 (0.580, 2.456)	0.592	1.000 (0.679, 1.471)	0.999	0.899 (0.547, 1.478)	0.663	0.694
CEMA	2.554 (1.027, 6.354)	0.045	1.511 (0.914, 2.499)	0.104	1.852 (1.044, 3.284)	0.036	0.602
CYMA	1.199 (0.807, 1.782)	0.327	1.324 (1.037, 1.691)	0.026	1.190 (0.866, 1.635)	0.270	0.981
DHBMA	4.019 (0.864, 18.695)	0.071	0.876 (0.347, 2.209)	0.772	1.415 (0.429, 4.671)	0.554	0.906
2HPMA	2.140 (0.730, 6.273)	0.144	0.943 (0.629, 1.415)	0.772	1.061 (0.729, 1.544)	0.749	0.975
3HPMA	2.077 (0.941, 4.584)	0.066	1.386 (0.806, 2.384)	0.229	1.742 (1.043, 2.909)	0.035	0.607
HPMMA	1.832 (0.808, 4.153)	0.129	1.545 (0.958, 2.491)	0.073	2.077 (1.044, 4.133)	0.038	0.527
MA	1.909 (0.554, 6.586)	0.268	1.109 (0.529, 2.327)	0.777	3.376 (1.561, 7.305)	0.003	0.137
MHBMA3	1.679 (0.853, 3.307)	0.118	1.333 (0.881, 2.017)	0.166	1.365 (0.807, 2.310)	0.233	0.962
PGA	2.311 (0.712, 7.502)	0.142	0.868 (0.394, 1.913)	0.717	0.626 (0.266, 1.471)	0.269	0.306

[a] Models were adjusted for age, race, smoker, physical activity, education level, drinker and marriage status. CI, confidence interval; OR, odds ratio; VOCs, volatile organic compounds.

Table S11. Sensitivity analysis of the relationship between urinary VOC metabolites and circadian syndrome by using PSM [a].

VOCs	Premenopausal		Postmenopausal	
	OR (95% CI)	*P*-value	OR (95% CI)	*P*-value
2MHA	0.988 (0.638, 1.531)	0.955	0.891 (0.690, 1.151)	0.369
3MHA+4MHA	0.823 (0.529, 1.281)	0.366	0.936 (0.702, 1.247)	0.644
AAMA	0.985 (0.584, 1.659)	0.951	1.424 (0.995, 2.037)	0.053
AMCC	1.342 (0.680, 2.651)	0.374	1.307 (0.893, 1.912)	0.164
ATCA	1.529 (0.848, 2.756)	0.147	0.909 (0.666, 1.240)	0.539
BMA	0.952 (0.582, 1.558)	0.836	0.937 (0.697, 1.259)	0.659
CEMA	1.894 (1.085, 3.306)	0.027	1.862 (1.343, 2.581)	< 0.001
CYMA	1.140 (0.886, 1.467)	0.288	1.242 (1.032, 1.495)	0.023
DHBMA	0.881 (0.302, 2.576)	0.807	1.467 (0.702, 3.063)	0.301
2HPMA	1.001 (0.557, 1.800)	0.998	1.085 (0.844, 1.395)	0.514
3HPMA	1.230 (0.733, 2.063)	0.410	1.598 (1.195, 2.137)	0.002
HPMMA	1.244 (0.707, 2.190)	0.427	1.746 (1.218, 2.504)	0.003
MA	0.146 (0.033, 0.636)	0.014	2.031 (0.835, 4.941)	0.115
MHBMA3	1.106 (0.649, 1.885)	0.694	1.373 (1.015, 1.856)	0.040
PGA	0.606 (0.271, 1.354)	0.206	0.886 (0.512, 1.534)	0.659

[a] Models were adjusted for age, race, poverty income ratio, smoker, physical activity, education level, drinker and marriage status. CI, confidence interval; OR, odds ratio; PSM, propensity score matching; VOCs, volatile organic compounds.

Table S12. Sensitivity analysis of the relationship between urinary VOCs and circadian syndrome after excluding individuals with urinary creatinine >300 mg/L or urinary creatinine <30 mg/L[a].

Circs	Premenopausal		Postmenopausal	
	OR (95% CI)	*P*-value	OR (95% CI)	*P*-value
2MHA	1.026 (0.808, 1.303)	0.831	0.827 (0.641, 1.066)	0.138
3MHA+4MHA	0.917 (0.698, 1.205)	0.527	0.885 (0.663, 1.181)	0.397
AAMA	0.933 (0.496, 1.755)	0.825	1.450 (1.020, 2.059)	0.039
AMCC	1.233 (0.740, 2.056)	0.413	1.286 (0.896, 1.847)	0.168
ATCA	1.536 (0.943, 2.504)	0.084	0.987 (0.713, 1.367)	0.935
BMA	0.741 (0.523, 1.050)	0.090	1.003 (0.768, 1.309)	0.984
CEMA	1.634 (1.108, 2.409)	0.014	1.778 (1.314, 2.407)	< 0.001
CYMA	1.074 (0.878, 1.315)	0.478	1.250 (1.045, 1.495)	0.016
DHBMA	0.838 (0.263, 2.678)	0.762	1.290 (0.619, 2.687)	0.488
2HPMA	0.688 (0.445, 1.063)	0.090	1.065 (0.807, 1.406)	0.650
3HPMA	1.050 (0.743, 1.483)	0.780	1.540 (1.132, 2.094)	0.007
HPMMA	1.118 (0.767, 1.629)	0.555	1.658 (1.161, 2.368)	0.006
MA	0.110 (0.014, 0.861)	0.036	1.364 (0.680, 2.737)	0.373
MHBMA3	1.058 (0.766, 1.462)	0.727	1.333 (0.994, 1.788)	0.055
PGA	0.551 (0.268, 1.133)	0.103	0.972 (0.561, 1.686)	0.919

[a] Models were adjusted for age, race, poverty income ratio, smoker, physical activity, education level, drinker and marriage status. CI, confidence interval; OR, odds ratio; PSM, propensity score matching; VOCs, volatile organic compounds.

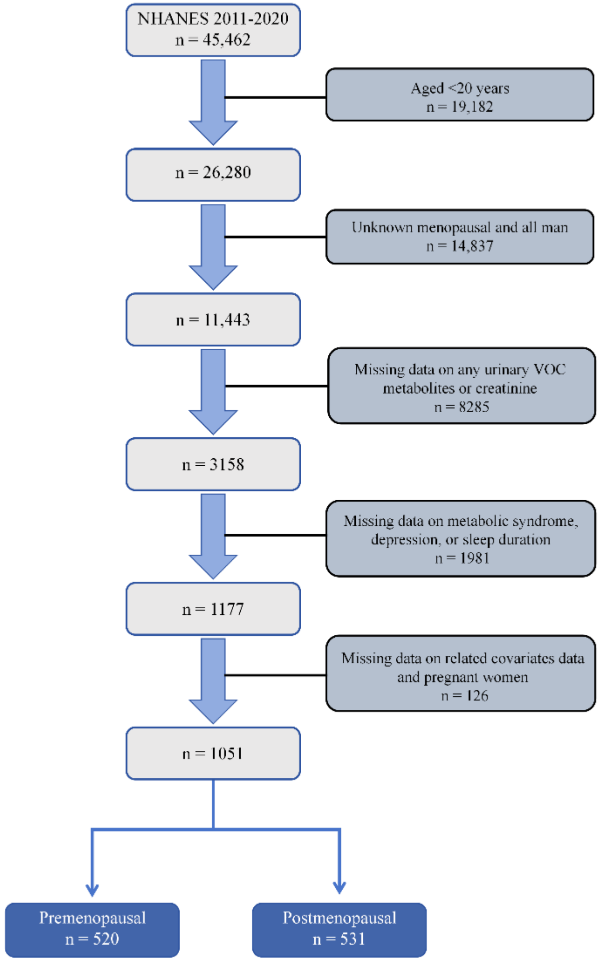

Fig. S1. Flowchart of participants included in this study. VOC, volatile organic compounds.

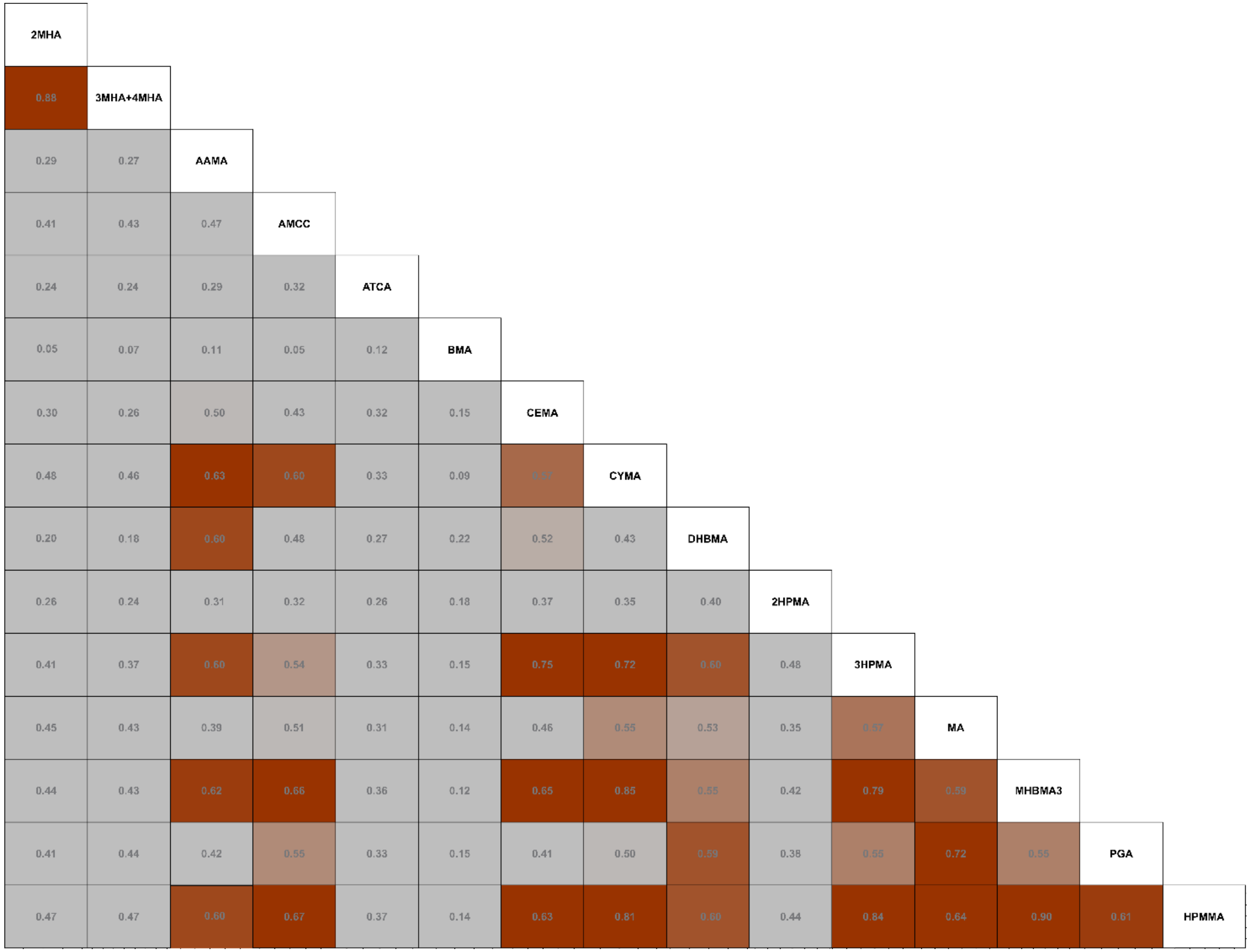

Fig. S2. Pearson correlations among the urinary VOC metabolites. VOC, volatile organic compound.

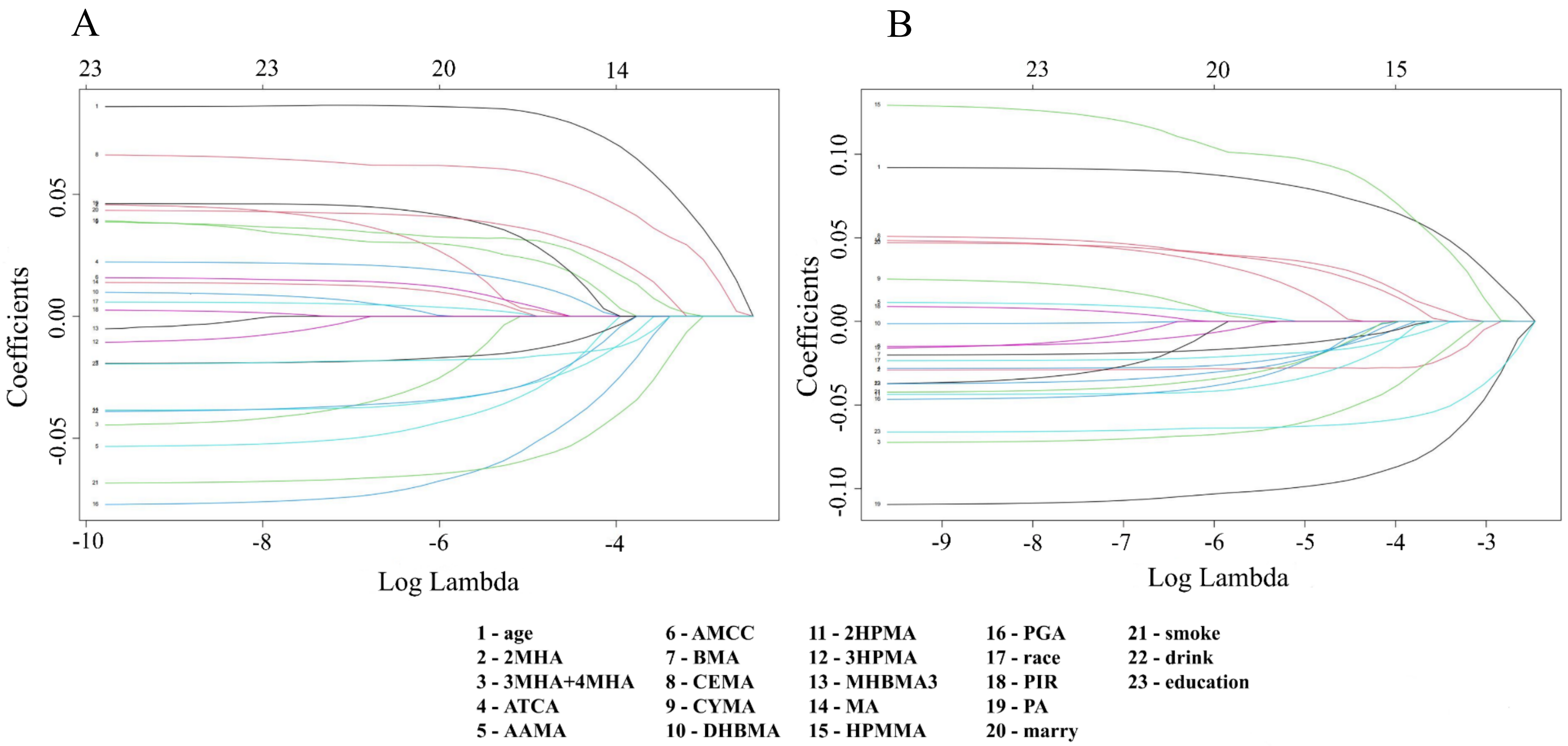

Fig. S3. Shrinkage coefficient curves between the log-transformed LASSO penalty parameter (λ) and the variates in premenopausal (A) and postmenopausal (B). The independent variables are ln-transformed 15 VOCs. Covariates include age, race, poverty income ratio, smoker, physical activity, education level, drinker and marriage status. LASSO, least absolute shrinkage and selection operator; VOCs, volatile organic compounds.

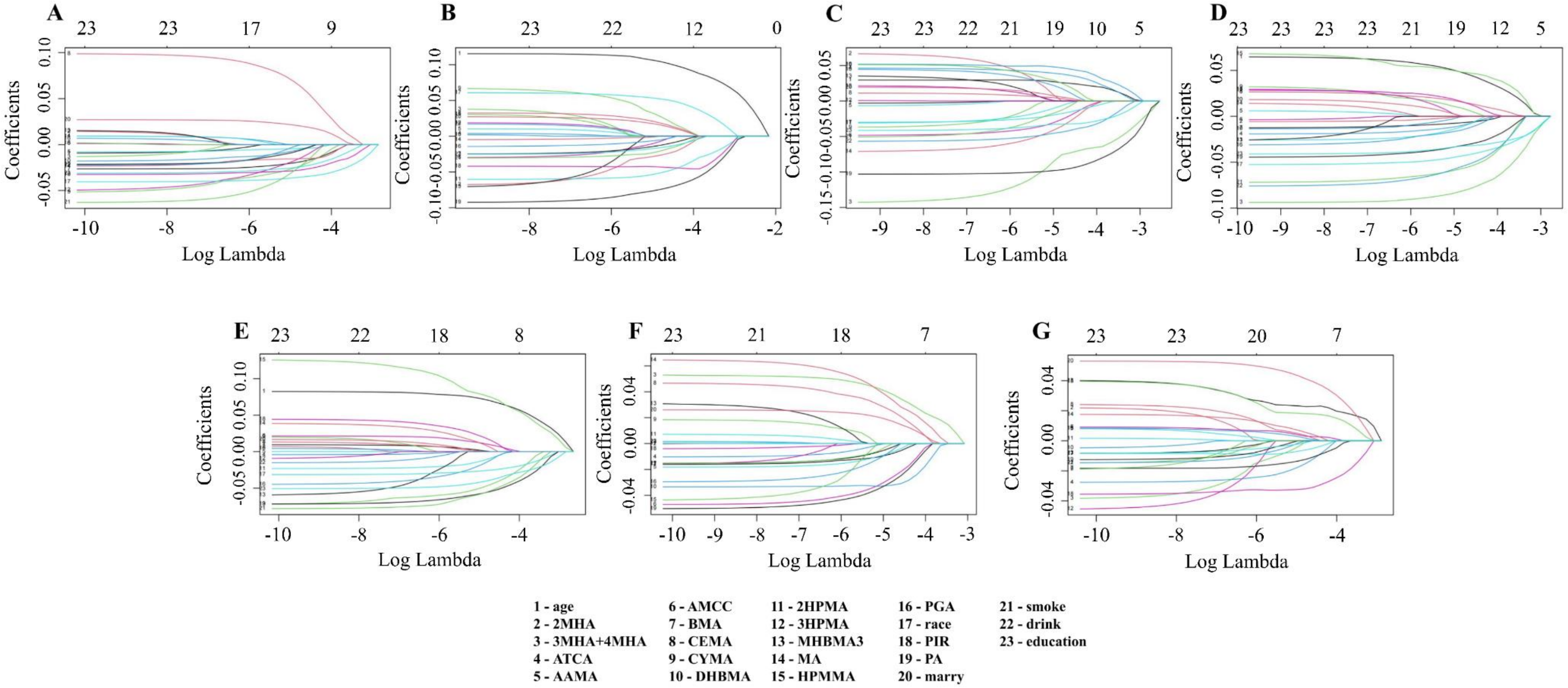

Fig. S4. Shrinkage coefficient curves between the log-transformed LASSO penalty parameter (λ) and the variates. The independent variables are ln-transformed 15 VOCs. Covariates include age, race, poverty income ratio, smoker, physical activity, education level, drinker and marriage status. Central obesity (A), Elevated blood pressure (B), Elevated glucose (C), Reduced HDL-C (D), Elevated triglycerides (E), Short sleep (F), Depression symptoms (G) were studied as dependent variables, respectively. HDL-C: high density lipoprotein cholesterol; LASSO, least absolute shrinkage and selection operator; VOCs, volatile organic compounds.

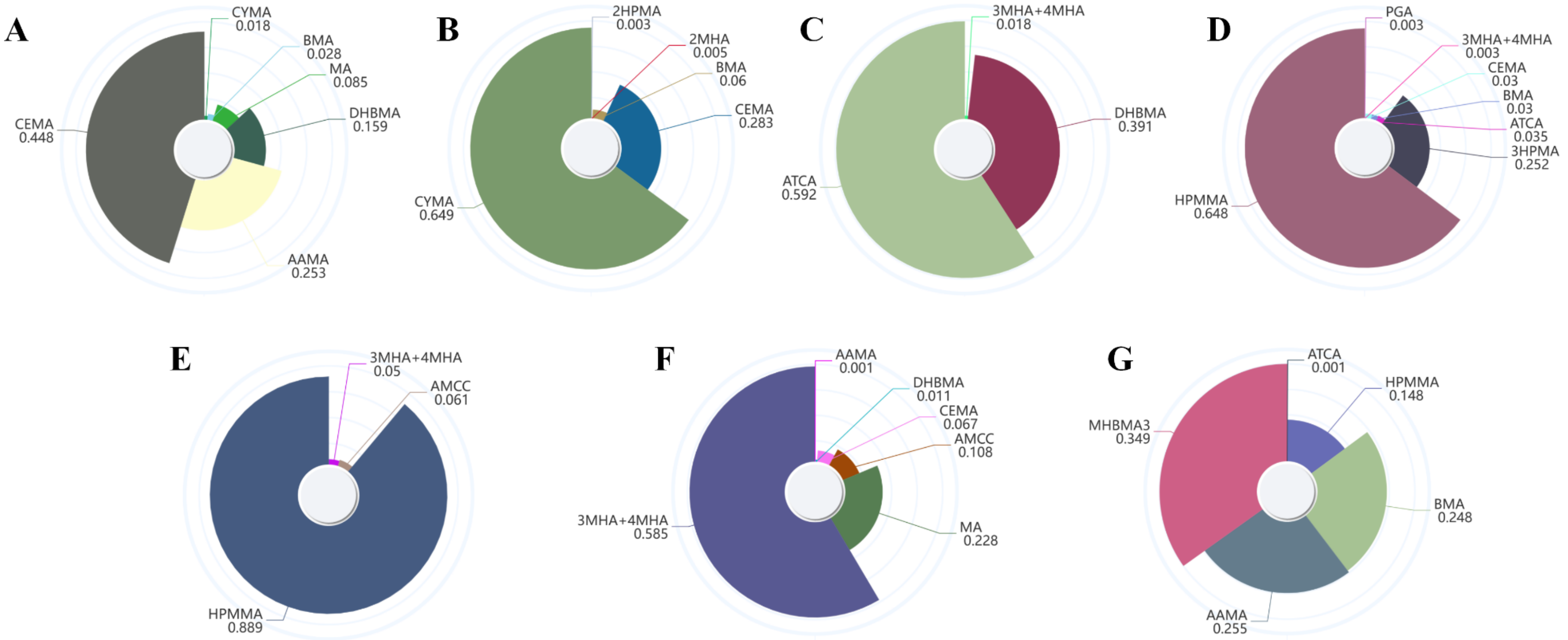

Fig. S5. The WQS model weights of screened urinary VOC metabolites on Central obesity (A), Elevated blood pressure (B), Elevated glucose (C), Reduced HDL-C (D), Elevated triglycerides (E), Short sleep (F), Depression symptoms (G). Models were adjusted for include age, race, poverty income ratio, smoker, physical activity, education level, drinker and marriage status. HDL-C: high density lipoprotein cholesterol; VOC, volatile organic compound; WQS, weighted quantile sum.
